# Data Summarization in the Node by Parameters (DSNP): Local Data Fusion in an IoT Environment

**DOI:** 10.3390/s18030799

**Published:** 2018-03-07

**Authors:** Luis F. C. Maschi, Alex S. R. Pinto, Rodolfo I. Meneguette, Alexandro Baldassin

**Affiliations:** 1 Department of Statistics, Applied Mathematics and Computing, Institute of Geosciences and Exact Sciences, UNESP—University Estadual Paulista, Rio Claro 13506-900, Brazil; alex@rc.unesp.br; 2 Department of Informatics and Statistics, Federal University of Santa Catarina, Florianopolis 88040-900, Brazil; a.r.pinto@ufsc.br; 3 Computer and Networking, Federal Institute of São Paulo (IFSP), Catanduva 15808-305, Brazil; meneguette@ifsp.edu.br

**Keywords:** data fusion, summarization, Internet of Things, IoT

## Abstract

With the advent of the Internet of Things, billions of objects or devices are inserted into the global computer network, generating and processing data at a volume never imagined before. This paper proposes a way to collect and process local data through a data fusion technology called summarization. The main feature of the proposal is the local data fusion, through parameters provided by the application, ensuring the quality of data collected by the sensor node. In the evaluation, the sensor node was compared when performing the data summary with another that performed a continuous recording of the collected data. Two sets of nodes were created, one with a sensor node that analyzed the luminosity of the room, which in this case obtained a reduction of 97% in the volume of data generated, and another set that analyzed the temperature of the room, obtaining a reduction of 80% in the data volume. Through these tests, it has been proven that the local data fusion at the node can be used to reduce the volume of data generated, consequently decreasing the volume of messages generated by IoT environments.

## 1. Introduction

In the last decade we have witnessed a new revolution in the era of information, not only compared with the emergence of the Internet itself in the 1990s, but also compared to the industrial revolution in the nineteenth century [1]. This new revolution is commonly known as the Internet of Things (IoT) or Internet of Objects, which basically refers to the connection of everyday objects to the world wide web, the Internet, usually with some ubiquitous intelligence [2].

One of the challenges of this new revolution is the sheer volume of data that the information age will generate [2,3]. One of the primary ways to help reduce this large amount of data is through a technique described as data fusion [4,5]. Among the different forms of data fusion, we mention the summarization of data [6], which works by extracting values, such as average, minimum, maximum, and totals of a magnitude read by a sensor or a group of sensors. Furthermore, data summarization also helps reduce the energy consumption in wireless sensor networks (WSNs) [7] and, as a consequence, in IoT environments. 

The purpose of this work is to perform the summarization of the data in the node (local data fusion), but through parameters provided by the server of an IoT (IoT Server) application, working in a combined way between sensor node and application server, called here *Data Summarization in the Node by Parameters* (DSNP). A significant advantage of this technique is that, through the parameters provided by the application stored in a server, the node can perform a more consistent data fusion process, seeking to ensure the quality of the data collected.

Therefore, a decrease in the volume of messages sent and, consequently, a smaller amount of data stored in the application server is expected, as well as lower energy consumption, but with a guarantee of the quality of the information collected. Currently, Internet traffic consumes 5% of all of the world’s energy in data transmission and processing [8]. With predictions of up to 50 billion devices being connected in the world-wide computer network [9,10], the energy consumption will increase considerably. Another point is the transmission of data in IoT environments, where a significant part occurs through the Wi-Fi protocol IEEE 802.11 [11], but may also use 3G and 4G cellular communication. The summarization process makes use of the values read from a database for the type of summarization, such as minimum and maximum values, and decides locally (at the node level) to perform the recording of the summarized values or not through a method known as Bollinger bands [12,13].

The main contribution of this article is to demonstrate that the data summarization through local data fusion in the node can contribute to the reduction of the data transmitted over the Internet, reducing the volume of data stored in the application and, consequently, reducing energy consumption in transmission, storage, and processing. The summarization process, even though it can lead to data loss, when properly performed, as will be demonstrated in the results section, can cause a decrease of the volume of data, while maintaining their quality and reliability. Our experimental results using two types of sensors reveals that DSNP can achieve a high reduction in the number of records stored in a server when compared to the traditional approach. In particular, a 97% decrease was observed for luminosity sensors, and 80% for temperature and humidity sensors. The technique developed in our work can be used in IoT or WSN applications. The present proposal was developed in a non-critical environment, not being tested and/or validated in critical applications, where several characteristics must be determined to be able to say that this process can be used in such a system. Therefore, the use of data summarization in critical environments may be the goal of future works. To the best of our knowledge this is the first time a local data fusion for IoT has been used to reduce the amount of transmitted data.

This article is organized as follows: In Section 2, concepts and techniques used in this work and related works are described. Section 3, will show how this work was developed and the functioning of the proposed algorithms. Section 4 presents the results and discussions about the values obtained in the tests performed. Finally, Section 5 presents the conclusions.

## 2. Background and Related Works

In this section we will explain the main methods used in this work, such as data fusion and the concept of summarization, the techniques of Bollinger bands for deciding whether or not to record summarized data, and a description of works more closely related to ours.

### 2.1. Data Fusion

There are several definitions in the literature for the concept of data fusion, being many times similar, but with some details and specificities according to some authors [10,11,12,13,14]. One of the most accepted definitions of data fusion is the combination of data from several sources aiming at an improvement in the final information, being it with higher quality, cheaper or of greater relevance [15]. The errors, the loss and the redundancy of the data in the transmission generate a great amount of problem at the moment of the interpretation of this data, causing disorders in the moment in which decision making becomes necessary. The data fusion itself is the application of techniques that interpret the data received by sensors scattered in a multi-sensor environment and analyze their validity and reliability [15].

Data fusion is used in many areas, such as robotics, artificial intelligence, image processing, in addition to being widely used in WSNs where it is extremely necessary, seeking to reduce costs and guarantee the information collected and, consequently, its use in IoT [16]. The concept involves several processes and techniques that seek the collection, verification, manipulation, combination, elimination, and grouping of data to obtain the necessary information [13,14,15,16,17]. These processes and techniques are used and applied at various moments, from the collection of the information in the node, transmission through various communication protocols, storage and until the moment of availability [18,19]. Data aggregation and data fusion [4] on sensors can help to compact and control the collected data in IoT environments. The concept of data fusion is widely researched in WSN, serving as one of the main points of support for the development of the IoT concept [20].

The algorithms that perform these techniques are called distributed data fusion algorithms [5], because they perform data fusion in a distributed way in the nodes or in gateways that perform the communication between the nodes and servers where the data will be stored [7]. The process of aggregation, which uses values such as mean, sum, minimum, and maximum, is known as data summarization [6]. Other data fusion mechanisms are proposed to assist in reducing the volume of data, eliminating redundancies, and organizing the applications so that they are managed in a way that makes the best use of a system of sensors for monitoring environments [21]. Data fusion proposals for monitoring complex environments, such as smart cities for example, are designed to seek better information quality and, consequently, better decision-making by the areas involved [21,22].

In WSN, data summarization [23] seeks to reduce the volume of messages that a WSN produces by combining data that belong to the same set of information collected by sensors of the same type. These data are combined and further summarized within the WSN and sent to a base station, which performs the final processing. Due to the low storage and processing capacity of sensor nodes, the use of more complex data fusion algorithms based on bio-inspired algorithms, such as neural and evolutionary networks [15,18], is sometimes prohibitive.

The fusion in the sensor node [24] allows for optimization of the data transmission. The possibility of using local data fusion executed in the node, in this case, more specifically, the summarization, is an opportunity that can be explored. Therefore, the goal of DNSP is to reduce the volume of data generated and, consequently, increase the energy savings with the transmission and better use of storage spaces in cloud computing environments.

### 2.2. Bollinger Bands

Bollinger bands, or Bollinger analysis, is the concept used for analysis of fluctuation of values within a given range, directly dependent on the arithmetic moving average (AMA) [13].

The moving average is the constant updating of values that form a given mean. This update consists of a number of fixed values, or vector, which generates a mean. As new values are inserted into the vector, the oldest values are discarded, keeping the number of vector elements always the same, but with the values updated.

Bollinger bands use the vector moving average to calculate a tolerable range, both above and below the mean. These above and below average values are known as Bollinger bands. To arrive at this range of values, we first calculate the mean and then the corresponding standard deviation. Having the standard deviation value calculated, we add in the AMM (arithmetic mobile media) plus two times the standard deviation for the upper edge calculation. For the lower band, we decrease the AMM value by twice the value of the standard deviation.

In this work, Bollinger bands are used by the algorithm for deciding whether to record the summarized values or not. If the values are within the tolerance range, in the case of the lower band and the upper band, the values are not recorded. If these values extrapolate these ranges, the values are recorded.

### 2.3. Related Work

A process of data fusion in the node can be observed in the work that seeks the detection of fire by multi-sensor methods [25], where luminosity, flame, and temperature sensors are used to detect fire using the arithmetic mobile media concept. A node stores the values of the *N* readings of the sensor, and calculates the average of these values. If the new reading is higher than the average value, the fire alarm is triggered. The use of multi-sensors (flame, temperature, and brightness) seeks to identify the possibility of fire, not generating false alarms if only a single sensor reads above average values. This approach, however, does not intend to store the collected data, but only focus on the decision-making (fire detected or not) by the mobile node.

The proposal of a system that employs local storage, Cloud Computing and the fusion at the local gateway is presented in the Smart e-Health Gateway project [26], where data collected by sensors from a particular location, a hospital for example, is analyzed by the gateway and the merge is performed locally in this gateway. Only the most relevant data are stored in the database on the Internet, and local officials have access to the entire information collected by the sensors regarding the patient. This approach is interesting in terms of local access to data, very useful in an e-Health system, but in applications where local storage is not possible and/or relevant, this approach becomes complex and has a higher cost.

The work closest to ours was proposed by Mohomed et al. [27], named the HARMONI platform (Healthcare-oriented Adaptive Remote Monitoring), developed more specifically for health support. The main idea is to use standard values in patient monitoring to ensure that the collected data is transmitted only when it is out of a given range. The example demonstrated in the tests was mainly cardiac frequency, and the main proposal is to send data only when these values are outside the specific standard range of each patient.

The prototype developed by Mohomed is composed of three layers. The first is the part of the patient’s sensing, using body sensors that, in the case of tests, verified the rhythm of the patients heartbeat and that formed a body area network (BAN) [27]. The second layer uses an application in which sensor data is received on a tablet and relayed to a server. The third layer is composed of the application where the data is stored and the standards of each user are defined by the responsible health professional.

HARMONI uses two communication protocols: the communication between the sensors and the tablet carried out by the Bluetooth communication protocol 1.2, based on the IEEE 802.15.1 protocol, and the communication between the tablet and the application server performed with the IEEE 802.11 protocol b/g. The proposal is to use a conscious context filtering, where the data and values within the desired standards for that patient are not transmitted, and only values outside this range are transmitted, such as cardiac arrhythmias or pulse oximetry values. The variables monitored on the patient through sensors are transmitted to the tablet, and the application configured in this tablet sends the data to the application server according to the parameters specified by the application. 

The main characteristics of the HARMONI project are the transmission of data only when the values are out of the stipulated standards, and the focus is on health support. To monitor environments where the variables that permeate their use change more consistently, the need to identify these changes is necessary, and a single system based on non-standard analysis values does not apply or can cause a loss of quality of the recorded data. It is necessary that, in addition to the non-standard data, the values read in a period of time are correctly recorded, thus guaranteeing the quality of data and a possible analysis for future decision-making.

The proposal of the use of multi-sensors to detect changes in the environment [25], seeking to prevent the occurrence of fires, seeks to merge data between information from more than one sensor, improving and guaranteeing the quality of the data collected, but the sensor node only triggers the alert when the event occurs. It is not possible to have a history of variations of the monitored environment.

The proposal of a local and cloud storage environment for a healthcare system [26] involves a more complex implementation process, involving local databases and cloud storage.

Finally, the proposal of the application HARMONI [27], seeks a solution where the fusion of data occurs in a tablet, this being the main point between sensors and data stored in a server, necessitating in this case a hub point.

In view of these problems, the present work was developed, in which we search for ways to reduce the volume of data through the development of an algorithm that performs the data summarization, thus guaranteeing data quality.

## 3. Data Summarization in the Node by Parameters

In this section we will discuss the application proposal, *Data Summarization in the Node by Parameters* (DSNP), the implemented algorithm for the data summarization. We also discuss the process of node summarization, the analysis of the need to record the data or not through the Bollinger bands and the roles of the database in providing the updated parameters, as well as storing data.

### 3.1. Proposal 

**Figure 1:**
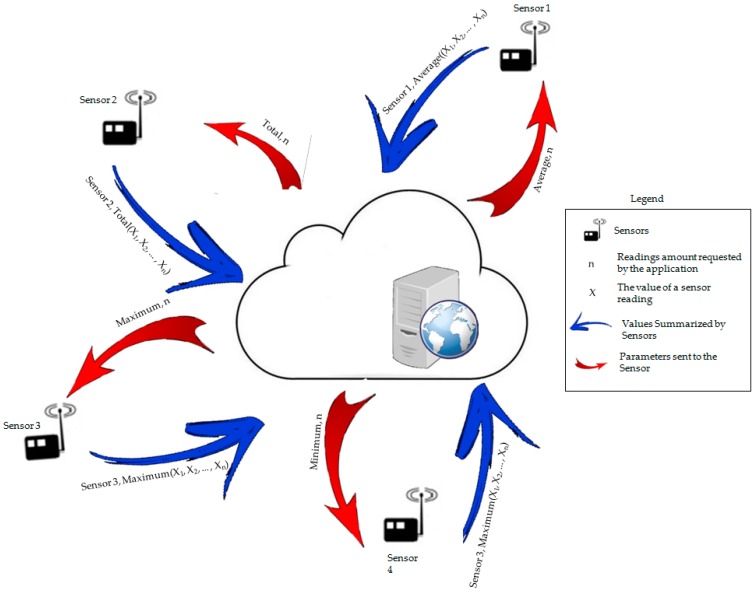
DSNP proposal.

The proposed architecture is an environment where the data will be stored, eventually, in a cloud computing environment. In this setting, the node, while connected to the application server, read the required parameters to perform the data summarization, as exemplified in Figure 1. Basically, the node, identified by an *id* will ask what type of summarization to perform and how many readings it should take to perform this summarization. After the summarization is performed, the node sends the data to be written to the application database.

The application is composed by an algorithm implemented in the node, which performs a query in a database, and returns the parameters that must be used by the node sensor. In turn, the database provides the parameters and receives the data read by the nodes, carrying out adjustments for some parameters that are then sent back to the nodes.

The choice of a hybrid system, where the vast majority of data fusion occurs at the node, but the information and parameters are provided by the application (usually in the cloud), leads to a reduction of the network traffic and, consequently, a decrease in the volume of data that needs to be stored. That happens because data is acquired based on parameters extracted from a history stored in the application server, which cannot occur on the nodes because of their low data storage capacity.

As an example, consider the case of the minimum and maximum values, where the initial values are specified empirically in the application by some user who has the knowledge and/or interest in these values in the application. Subsequently, as the values are inserted, we can extract minimum and maximum values obtained in searches in the database with the actual values read by the sensors, such as the minimum temperature of the last month or the same month of previous years. Parameter values are then based on data that will be stored in the application itself.

This solution also provides an option for changing the parameters dynamically. For example, if a certain sensor is obtaining the average luminosity of a given classroom in 5-min periods but, rather than the average, it is detected that the highest luminosity value is required over a period of one minute, this change may occur in the application which, after being read by the node at the time of recording, or when restarted, will provide the information according to the specified information.

**Figure 2:**
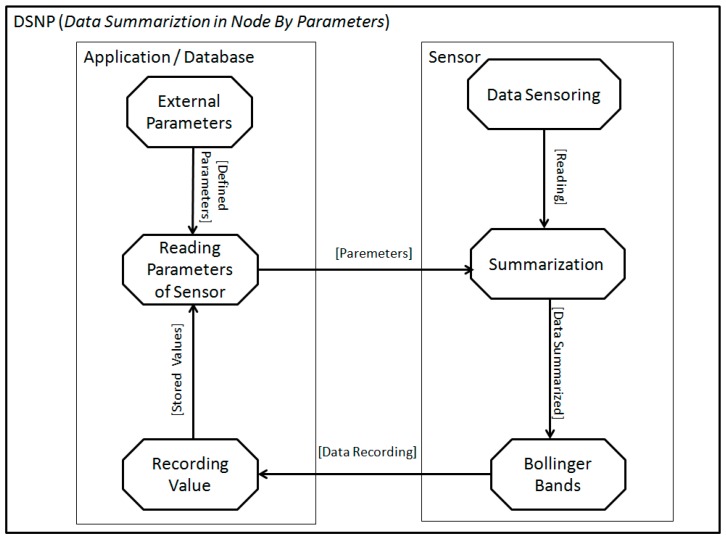
The application proposal using Data Summarization in the Node by Parameters (DSNP).

In Figure 2 we observe a DSNP operation, divided into two parts, the sensor and the application/database. The sensors part has processes for data sensing, summarization, and Bollinger bands. The process of data sensing refers to the collection of data by the node through the sensors, later sending these values so that the summarization process performing the summarization of the data. Finally, the process of Bollinger bands verifies the need to record the data, or not, in the application database. The application/database has the processes of receiving and storing external parameters, the process of recording value, responsible for storing the data sent by the sensors node and, finally, the process of reading parameters of the sensor, which performs the reading of the external parameters supplied by the application together with the values already stored by the sensor nodes, sending this values to the sensor node when requested.

### 3.2. Algorithm Summariztion

**Figure 3:**
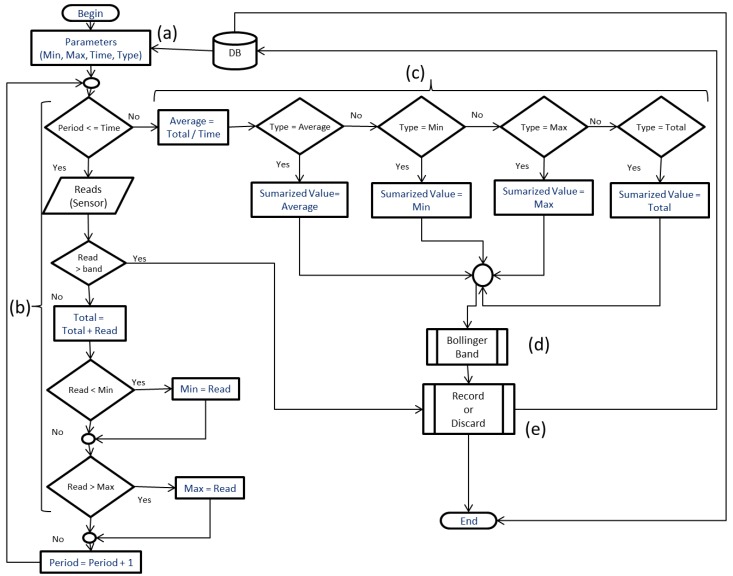
The proposed summarization algorithm in the node.

The operation of the algorithm in the node sensor basically makes a query to a database, searching the collection period, the type of summarization that was defined in the application (average, minimum, maximum, total, etc.), and the respective values, as observed in Figure 3a. After that, the data is summarized (Figure 3b), obtaining the minimum, maximum, and total values of the readings performed during the time according to the database. When the number of readings is finalized, the type of summarization defined in the database is verified (media, minimum, maximum, total) by recording the value according to the summarization defined in the application (Figure 3c). Next, the Bollinger bands (d) are analyzed to decide whether to record the data or not (by sending it to the database). If the return is positive, the value is recorded in the database, otherwise the value is discarded (Figure 3e). This algorithm demonstrates the implementation of summarization in the node through the method of aggregation of distributed data, using minimum, maximum, total, and average values to accomplish the summarization of the data.

The method of summarization is not combined, but chosen among the types of summarization, such as the average, minimum, maximum, and total. Thus, according to the characteristic or necessity of reading a certain value of that environment, the user or application can choose the best type of summarization for that type of value.

The minimum and maximum values allow the data to be sent directly to the server in case the respective readings are not within the application-stipulated values. In the server, the stored data can be used as a way of identifying possible changes in the environment that somehow affect the elements involved.

In case of large variations between readings, the node performs a simple check, where if the previous value compared to the current value exceeds a stipulated difference, the algorithm reads the records value for the identification of sudden variation in the environment. The procedure to identify these variations is relatively simple, where the difference between the minimum and maximum values is obtained and, from this value, a percentage of 10% difference is verified. This percentage of 10% was defined empirically. If the difference in readings between the previous value and the current value exceeds this percentage, the node sends the data to be recorded in the database. For example, if a node has minimum and maximum values between 22 °C and 32 °C (degrees Celsius), a difference of one degree Celsius is enough for the node to send the reading to the server and the application performs the stipulated procedures for the decision-making.

### 3.3. Algorithm for Bollinger Bands

In the algorithm implemented in the node, the Bollinger bands technique is used to analyze whether the summarized values should be recorded in the database or not. The last *N* summarized values are allocated in a vector in the function that calculates the moving average and the Bollinger bands, shown in Figure 3d.

**Figure 4:**
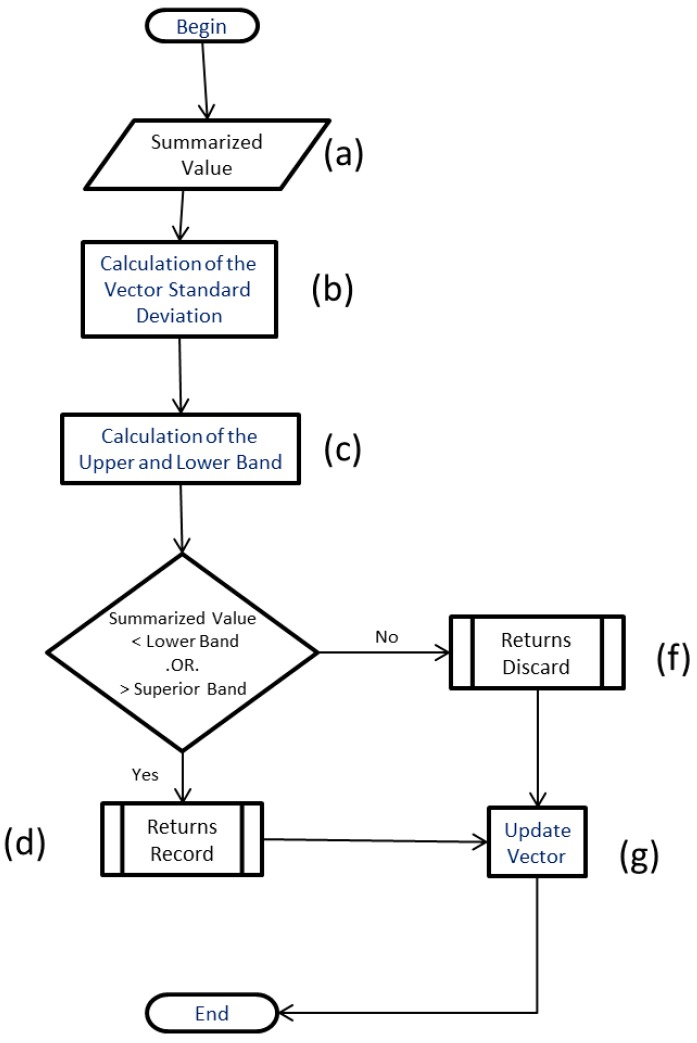
Bollinger bands in summarization algorithm.

At each summarization cycle, the algorithm sends the summarized value for Bollinger Band checking (Figure 4a), where the moving average is calculated (Figure 4b) and its upper and lower bands defined as Bollinger bands (Figure 4c), are identified. If the summarized value is within the bands, the main function returns the command for not recording the value in the database (Figure 4f). Otherwise, the value extrapolates the bands and the command sent is for the value to be recorded (Figure 4d). After the calculation is performed, the vector is updated (Figure 4g), discarding the oldest value, rearranging the index of the vector according to the entries, and the new value is inserted into the vector, working as a queue.

### 3.4. Application (Database)

The application has a database where there is a register of the nodes, which takes the name of the node, the type of magnitude monitored by the node, and a location. It also has the summarization type registration module, the initial time interval between sending the messages with the data to be recorded, in addition to the minimum and maximum, total or last values read values. This information is usually queried by the node at the time it is initialized or restarted.

**Figure 5:**
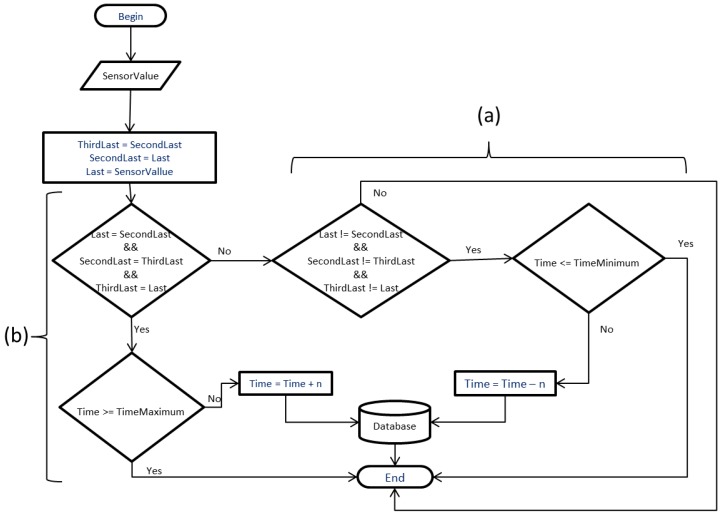
Update of the summarization times in the database.

Exemplified in the flowchart of Figure 5, the values recorded by the nodes are analyzed, and if the values do not change, the data send times are increased as demonstrated in Figure 5b. If these values change from one reading to the other, the time is maintained, and if the values change within three different readings, the data send time is reduced, as demonstrated in Figure 5a.

Another algorithm in the database verifies in a certain period of time, in the case of this work, every day, the maximum and minimum values read by the node stipulated empirically. The maximum and minimum values found in the time period of seven days are used as parameters, passed to the nodes to perform the summarization process, as long as these values do not overlap with the specified minimum and maximum values within the application. For example, if the minimum value of the readings recorded in the last seven days is greater than the minimum value stipulated in the application, the minimum value is changed according to the standard reading in this time period, which in this case is seven days even for the maximum value, provided that the read values are not greater than the stipulated maximum value. Notice that the summarization type is specified by the application.

## 4. Results and Discussions

In this section an evaluation of the proposed technique is carried out. We seek to directly evaluate the storage size compared to a typical system without data fusion.

### 4.1. Experimental Settings

Two sets of sensor nodes were configured, and each set was composed of two sensor nodes configured with the same equipment for reading (sensing) and data transmission. The difference between one node and the other was only the way the data was sent. The first node sent the data in a standard way, sequentially, performed the reading in a period of 10 s, which are then recorded in the database. The second node sends the summarized data following our previously-described approach (DSNP).

The first set of nodes was configured with two sensor nodes composed of a NodeMCU platform [28] with a DHT11 temperature and humidity sensor (digital humidity and temperature) that receives the data on a digital port. These nodes were allocated side by side in one of the teaching labs. It needs to be emphasized that this environment has several application servers, where some work tools are configured to teach and research in the area of networks, thus requiring a controlled temperature so that the equipment and servers that support these applications do not suffer overheating. It has been stipulated that temperatures should range between 18 °C and 28 °C.

The other set of nodes configured was comprised of a NodeMCU platform [28] and a light-dependent resistor (LDR) light sensor, receiving the values on an analog port. Like the first set of nodes, one of the nodes sends the data continuously every 10 s, where the other employs our data fusion mechanism.

In the following sections, we use the term *standard recording* to refer to the default method of recording a sample every 10 s, and *DSNP recording* to refer to our approach. The tests were performed over a period of seven uninterrupted days. In the next two subsections the data recorded by each sensor set will be analyzed separately.

The first set of sensors, configured with the DHT11 sensor, had initial values between 18 °C and 28 °C as stipulated for the laboratory environment. In the set of sensors composed with the LDR sensors no minimum or maximum value was stipulated, these values being between 0 and 1023, due to characteristics of these sensors having a very large range of values.

### 4.2. Temperature Sensors

**Figure 6:**
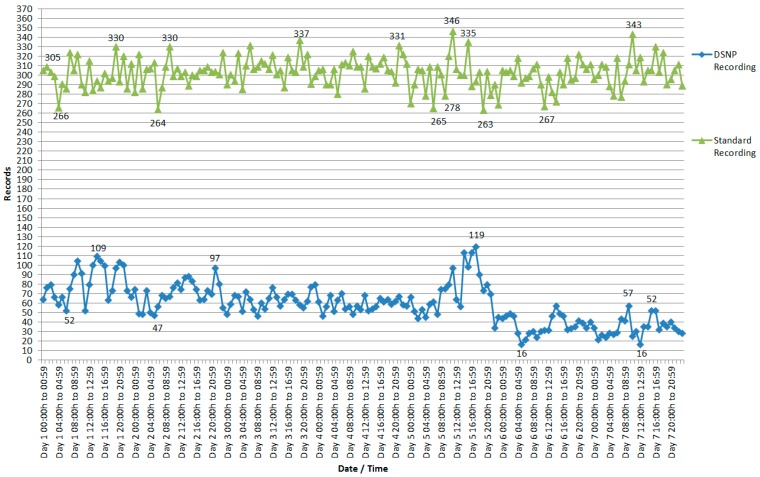
Number of records sent per each temperature sensor.

We first analyze the results given by the temperature sensors. Figure 6 shows the amount of records during a week for the two methods compared in this work. As can be seen, 50,808 standard recordings were performed sequentially, averaging 302 recordings per hour. The node executing with the DSNP approach performed during the same period of seven days made 9902 recordings, for an average of 59 recordings per hour. Compared to the standard recording, DSNP provided a decrease in the number of records made by 80.5%.

**Figure 7:**
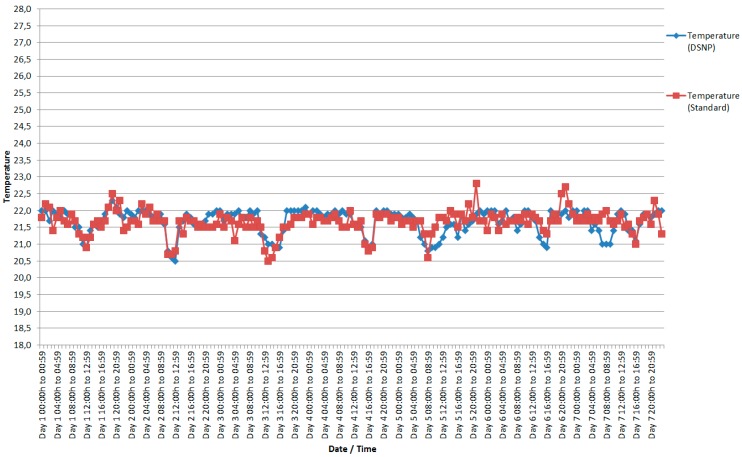
Average temperatures recorded by the standard shape sensors and average temperatures of the sensors recorded using DSNP.

In order to assess the quality of results of our DSNP approach, Figure 7 presents the temperature measurements for the considered week. As can be observed, the values are very similar, with a tenth of a degree difference in relation to the sensors for the nodes that sent the records sequentially. The average temperatures recorded in the standard form, sequentially identified in Figure 7 by *Temperature (Standard),* were very close to the average temperatures recorded by the nodes configured with the algorithms proposed in this work, identified in the same figure as *Temperature (DSNP).*

In Figure 7 it can be observed that the difference between the values recorded sequentially and the use of the DSNP method is relatively small, not reaching 1 °C. Notice, also, that the accuracy of the sensor used is low (DHT11) and the temperature’s granularity is one degree. For the type of monitoring that is proposed in this work, a school environment, it is a very small difference, not resulting in large issues for the application or the environment that is being observed. Obviously, in case of an environment that needs greater precision, another sensor should be used.

### 4.3. Luminosity Sensors

**Figure 8:**
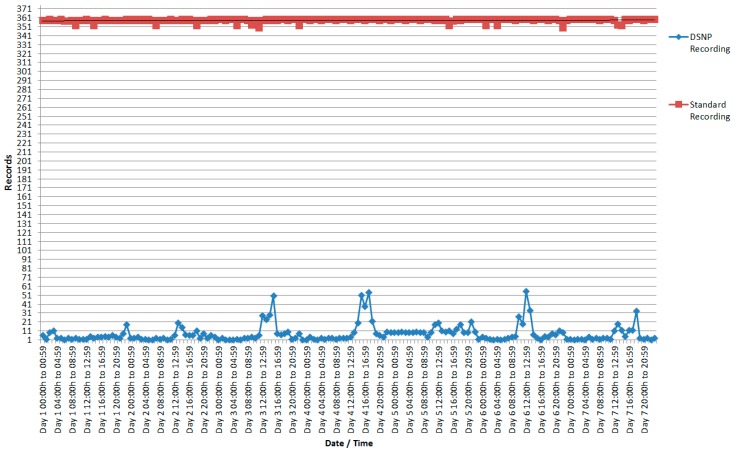
Number of records sent per each luminosity sensor.

**Figure 9:**
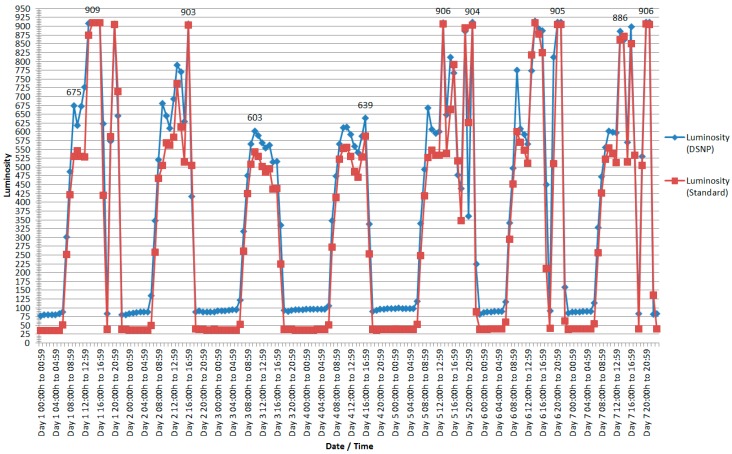
Average luminosity recorded by the standard shape sensors and average luminosity of the sensors recorded using DSNP.

In the second experiment, we analyze the values of the set of sensor nodes that measure the brightness of the classroom, shown in Figure 8 (records) and Figure 9 (luminosity). 

As a first observation, the large difference presented in the number of registers needed to obtain the luminosity stands out (Figure 8). Although the standard recording averaged 358 recordings per hour sequentially over a seven-day period, the DSNP approach averaged only 8 recordings per hour in the same period. In percentage terms, this corresponds to a reduction in the number of records of 97.77%. In total, the number of records stored by each sensor with standard recordings was 60,148 standard recordings and 1327 records recorded in summary form, using DSNP.

Regarding the data quality, it is observed in Figure 9 that the values were very close between the readings of standard form and using the DSNP approach. Note that the range of values measured by this type of sensor is large, where the value 0 (zero) is very close to the total darkness and the value of 1023 is the maximum luminosity collected by the sensor.

### 4.4. Comparison between Sequentially-Recorded Records and those Summarized by the Node

Table 1 presents the number of records for the two groups of sensors in the considered week, comparing the number of records acquired through the sequential and our DSNP methods. As we already pointed out in the previous sections, the DSNP approach, when compared to the sequential recording, required only 19.49% (temperature) and 2.18% (luminosity) of the total number of records.

Where the percentage refers to the number of records recorded in summary form in relation to the records recorded sequentially, for example, on Day 1, only 26.95% of the records were recorded in a summarized form by the node regarding temperature sensing of the network laboratory in relation to the node that performed the sequential sensing without any form of filter. Continuing the example, on the same day, the node responsible for sensing luminosity in the classroom in a summarized way performed only 1.29% of the recording of the records in relation to the node that made the recordings sequentially.

It can be observed in this context that although the reduction in data volume has been considerable, as shown in Figure 5 and Figure 7 and in Table 1, the quantities of registered records, data quality, and changes in the environment variables monitored by the nodes through the sensors continued to reflect what was happening in the environment consistently, as can be seen in Figure 6 and Figure 8. This demonstrates that data summarization processes at the nodes can still capture the real values of the environment, but more economically due to the reduction in the volume of data, reflecting indirectly on energy consumption.

## 5. Conclusions

With the growth of IoT and the use of sensors to monitor environments, the concern with the volume of generated data has increased. Data fusion is a good option to reduce such volume, with data summarization being of great help.

In this article, a proposal was made to use data summarization in the node through the parameters provided by the application. By means of these parameters, the sensors can perform a local decision as to whether the data should be sent to the server or not through the Bollinger bands technique. 

The results obtained in the tests were very satisfactory, having a reduction of around 80% in the case where the sensor requires a more precise range of values, such as temperature and humidity sensors. In sensors where a range of values read is wider, like a light sensor, the reduction reached up to 97% of the data. The DSNP approach was shown to be efficient in reducing the amount of generated data, being a viable option for reducing the volume of data generated in environments monitored by sensors.

## Figures and Tables

**Table 1:** Relation of recordings due to the sequential and DSNP methods by day.

Day	Temperature			Luminosity		
	DSNP Records	Sequential Records	% Difference	DSNP Records	Sequential Records	% Difference
1	1939	7196	26.95%	111	8583	1.29%
2	1661	7253	22.90%	121	8587	1.41%
3	1502	7381	20.35%	207	8580	2.41%
4	1406	7373	19.07%	264	8609	3.07%
5	1705	7107	23.99%	256	8602	2.98%
6	876	7198	12.17%	214	8588	2.49%
7	813	7300	11.14%	139	8599	1.62%
Total	9902	50,808	19.49%	1312	60,148	218%
